# Comparison of feeding habits and habitat use between invasive raccoons and native raccoon dogs in Hokkaido, Japan

**DOI:** 10.1186/s12898-019-0249-5

**Published:** 2019-09-11

**Authors:** Aya Osaki, Mariko Sashika, Go Abe, Kohei Shinjo, Ayako Fujimoto, Mariko Nakai, Michito Shimozuru, Toshio Tsubota

**Affiliations:** 1 Department of Environmental Veterinary Science, Graduate School of Veterinary Medicine, Hokkaido University, Kita 18 Nishi 9, Kita-ku, Sapporo, Hokkaido 060-0818 Japan; 2 Wildlife Research & Consulting Services Ltd, 94-2 Saji, Aogaki, Tamba, Hyogo 669-3811 Japan; 3 Shiretoko Nature Foundation, 531 Iwaubetsu, Shari, Hokkaido 099-4356 Japan; 4 Raccoon Researchers Group, Kita 21 Nishi 3, Kita-ku, Sapporo, Hokkaido 001-0021 Japan

**Keywords:** Invasive species, Raccoon, Native species, Raccoon dog, Feeding habit, Habitat use

## Abstract

**Background:**

In Japan, invasive raccoons cause severe ecological and social problems by transmitting pathogens to humans, livestock, and native species, causing substantial crop damage, and competing with native species. Possible competition between invasive raccoons and native raccoon dogs is of concern in Japan because Japanese raccoon dogs have a limited distribution and are native only to Japan and the two species have similar characteristics. We assessed potential competition between raccoons and raccoon dogs by comparing feeding habits and habitat use.

**Results:**

Both species were captured in Hokkaido, Japan from 2004 to 2017. More raccoons were captured close to agricultural land at the forest periphery (70.1%, 358/511); conversely, more raccoon dogs were captured in the forest core (74.9%, 253/338). Feeding habits were then examined by fecal analysis and stable isotope analyses. Fecal analysis revealed both species to be opportunistic omnivores that consumed easily found food items. However, raccoon feces contained more crops, whereas raccoon dog feces contained more insects, reflecting the different locations in which the species were trapped. Moreover, stable isotope ratios were significantly higher in raccoons than raccoon dogs (Corn has the highest carbon stable isotope (δ^13^C) value, and amphibians and reptiles are high in nitrogen stable isotope (δ^15^N); forest resources such as insects and wild fruits are low in δ^13^C and δ^15^N).

**Conclusions:**

We conclude that both species ate similar food types, but their food preferences appeared to differ. Raccoon and raccoon dog habitat use also differed, possibly because the two species inhabited areas where they could easily obtain their preferred foods. Therefore, the current feeding habits and habitat use of raccoons do not appear to overlap sufficiently with those of raccoon dogs to impact the latter. The results of this study, particularly the stable isotope data, may provide a useful precedent for future studies of competition in medium-sized mammals, particularly canids.

## Background

Raccoons (*Procyon lotor*) are medium-sized omnivores native to North America [1]. Because of the influence of the cartoon ‘‘Rascal Raccoon’’ on television in 1977, raccoons became popular pets in Japan, which encouraged substantial importation from North America [2]. Through intentional release/abandonment or escape, many non-native raccoons became naturalized in Japan [2]. Raccoons have now been confirmed in all Japanese prefectures, and they cause severe ecological and social problems [3], which include: (1) impacts on native ecosystems, such as competition with and predation on native species; (2) transmission of pathogens to humans, livestock, and native species; and 3) substantial crop damage [2]. Although research on raccoon infectious diseases has been carried out [4, 5], little research has evaluated the impact of raccoons on native ecosystems in Japan [6], because it is difficult to collect data on the effects of raccoons on native species [7].

Competition occurs when two or more species occupying the same habitat at the same time are using some environmental resource [8]. As noted by Kuzman [9], “if competition occurs, one or more of the competing species will suffer reduced fitness as a result of (1) less resources being available, and (2) energy used up in direct contest or interference with other competitors.” Consequently, native species become extinct or decline in population size and are replaced by the invasive species. A system for classifying alien taxa based on the magnitude of environmental impacts was developed by Blackburn et al. [10]. This classification system uses five semi-quantitative scenarios that describe the different levels of impact of a species—ranging from massive to minimal—with assignment corresponding to the highest level of associated deleterious impacts. Massive competition results in replacement or local extinction of one or several native species, and changes in community composition are irreversible. Major competition results in local or population extinction of at least one native species, which leads to changes in community composition, but changes are reversible when the alien species is removed. Moderate competition results in a decline of population size of at least one native species, but no changes in community composition. Minor competition affects fitness (e.g., growth, reproduction, defense, and immunocompetence) of native individuals without decline of their populations. Finally, minimal competition represents a negligible level of competition with native species, in which reduction of fitness of native individuals is not detectable [10]. To our knowledge, raccoons have not been classified in Japan according to competition risk.

Raccoon dogs (*Nyctereutes procyonoides*) are native to Japan and may be most at risk of direct competition with non-native raccoons [3] because both species are nocturnal, medium-sized, omnivorous forest inhabitants. Raccoon dogs are widely distributed throughout Japan, excluding Okinawa Prefecture. Of the two subspecies, *N. procyonoides viverrinus* occurs in Honshu, Kyushu, and Shikoku, and *N. procyonoides albus* occurs in Hokkaido [11]. Although raccoon dogs are common in Japan, little research has been performed on ecological characteristics of *N. procyonoides albus*. Despite Japanese raccoon dogs being considered a subspecies of the continental population, recent research has shown that Japanese raccoon dogs actually have morphological and molecular characteristics that differ compared with the continental population [12, 13]. Japanese raccoon dogs are indigenous to and only inhabit Japan. In particular, the *N. procyonoides albus* distribution is narrower and more limited than that of *N. procyonoides viverrinus.* Consequently, an ecological survey of *N. procyonoides albus* is needed, including an investigation of the influence of raccoons on raccoon dogs.

**Fig. 1:**
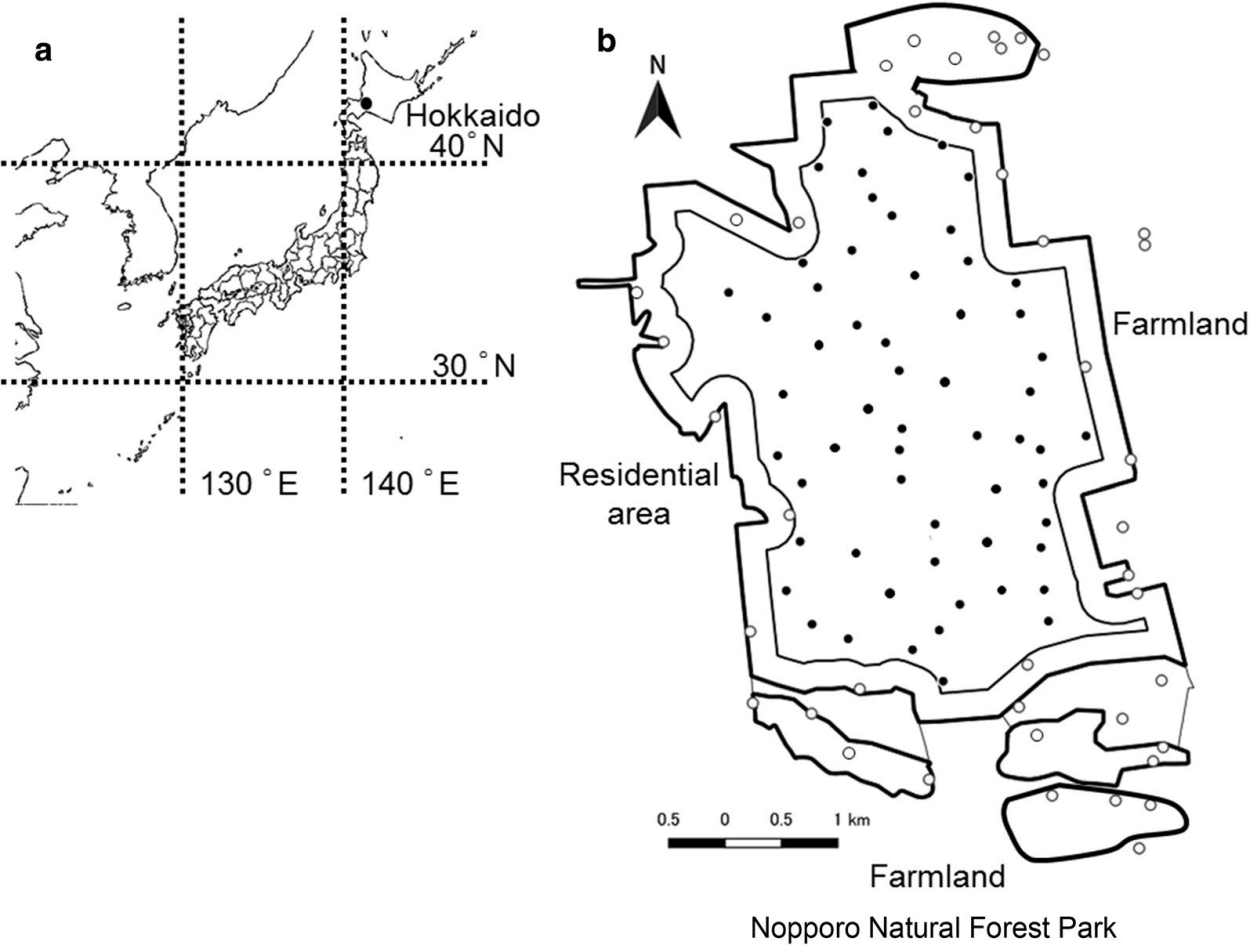
Sampling locations at Nopporo Natural Forest Park. **a** Map of Nopporo Natural Forest Park, central Hokkaido, Japan. **b** Map showing the trapping points in Nopporo Natural Forest Park. Open circles show the forest periphery (41 points) and closed circles show the forest core (59 points). This figure was developed using data from National Land Numerical Information (Administrative Zones, Natural Park) and edited by us (Publication of the figure under a CC BY license was permitted by the National Spatial Planning and Regional Policy Bureau, MLIT of Japan, copyright 1974–2018)

In the study area, Nopporo Natural Forest Park, we have been investigating raccoon dog and raccoon biology since 2004, when only nine raccoon dogs were captured. Mange may have been one cause of the low capture rate because mange was prevalent (Sashika M., personal observation). However, raccoon dog populations may have also decreased as a result of the influence of raccoon introduction. Understanding the potential impact of raccoons can help conservation practitioners make informed decisions to protect native wildlife. In this study, we examined the potential impact of raccoons on raccoon dogs by comparing their habitat use and feeding habits. To compare habitat use, we surveyed the capture sites of both species to assess whether their habitats overlapped (Fig. 1).

We used both fecal analysis and stable isotope analysis to determine the food items that each species ate. Fecal analysis allowed us to directly observe the food items each species had eaten. This method is noninvasive and has little influence on the behavior and ecology of the sampled animals [14], although it may be biased by the digestion rates of different food items. For example, fecal analysis may miss soft-bodied prey, such as amphibians, that were digested before analysis was performed [15]. For short study periods, fecal analysis may not adequately describe the long-term feeding habits of a species [15].

In contrast, stable isotope analysis of animal tissue may better characterize long-term feeding habits. Carbon (δ^13^C) and nitrogen (δ^15^N) stable isotope signatures in animal tissues provide information about diet, because the stable isotopes in the consumer’s tissues are related to those of its diet [16, 17]. The δ^15^N isotope signatures of species that are higher in the food chain have higher values than those of species lower in the food chain [17, 18]. Consequently, the δ^15^N isotope signature of a species may be used to estimate the trophic level within which a species exists. The turnover of stable isotopes in a particular tissue is related to the metabolic activity of that tissue [19]. Therefore, the isotopic signatures of different tissues from the same consumer can provide short- and long-term dietary information [20, 21]. We used hair from captured raccoons and raccoon dogs to analyze isotope signatures. Hair is particularly useful for this purpose, especially compared with tissues such as blood (red blood cells and plasma) and muscle. This is because hair archives temporal (seasonal) fluctuations in diet isotope composition, can be obtained in a noninvasive manner, and preserves diet information over time (although not during quiescent phases) [15].

The purpose of this study was to clarify whether habitat and food resources of raccoons and raccoon dogs overlap to help discover whether there might be resource competition between these species. We hypothesized that the habitat and food resources of raccoons and raccoon dogs would overlap. Our study provides novel information regarding the potential competition between an invasive species (raccoons) and a native species (raccoon dogs) in an isolated forest. Because invasive species have been increasingly problematic worldwide, the information in our study could be helpful for informing future research on the impact of invasive species on other canids worldwide.

## Results

### Numbers and locations of raccoons and raccoon dogs captured

**Fig. 2:**
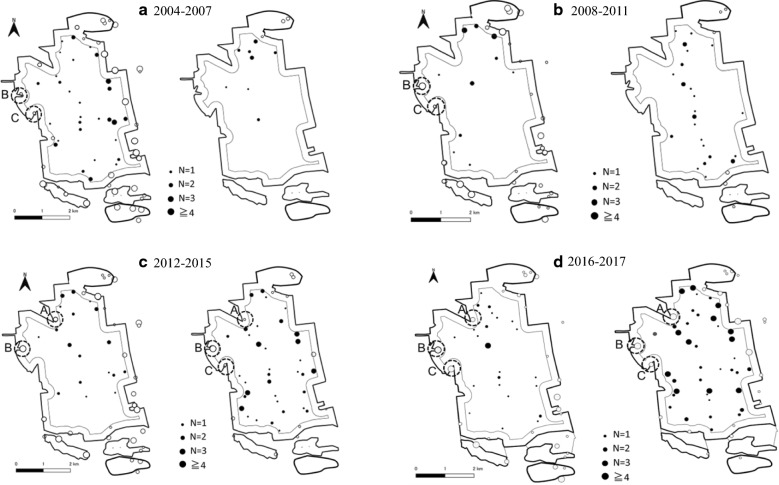
Capture sites of raccoons (left map) and raccoon dogs (right map) from 2004 to 2017. Open circles show the forest periphery; closed circles show the forest core. Circle size is proportional to number of raccoons and raccoon dogs captured. A, B, and C of the forest periphery indicate sites where many raccoons and raccoon dogs were captured. **a** 2004–2007, **b** 2008–2011, **c** 2012–2015, **d** 2016–2017. This figure was developed using data from National Land Numerical Information (Administrative Zones, Natural Park) and edited by us (Publication of the figure under a CC BY license was permitted by the National Spatial Planning and Regional Policy Bureau, MLIT of Japan, copyright 1974–2018)

The numbers of captured raccoons and raccoon dogs are shown in Table 1 (excluding juveniles). Between 2004 and 2017, the number of captured raccoons varied between 20 and 50 every year. The number of captured raccoon dogs was fewer than 17 from 2004 to 2013. The raccoon dog population has gradually recovered since 2014. The capture sites of both species are shown in Fig. 2. The capture sites of raccoons did not change much by year, and raccoons were often captured at the forest periphery. Most raccoon dogs were captured in the forest core by 2013, when the number of captured raccoon dogs was still low. Subsequently, as the number of captured raccoon dogs increased, the number captured at the forest periphery also gradually increased. In total, 70.1% (358/511) of raccoons were captured at the forest periphery, which is close to agricultural land, and 74.9% (253/338) of raccoon dogs were captured in the forest core (Fig. 2; Table 1). A Chi squared test showed that the number of raccoons captured at the forest periphery was significantly higher than that of raccoon dogs (P < 0.05). Conversely, the number of raccoon dogs captured in the forest core was significantly higher than that of raccoons (P < 0.05). There were three trapping points (A, B, and C) at the forest periphery near the urban area, and 12 raccoons and 14 raccoon dogs were captured between 2016 and 2017 (Fig. 2).

**Table 1:** Number of raccoons and raccoon dogs captured in Nopporo Natural Forest Park

	The capture site	2004–2007	2008–2011	2012–2015	2016–2017	Total
Raccoon	Forest core	57 (30.8%)	26 (28.0%)	37 (27.2%)	33 (34.0%)	153 (29.9%)
	Forest periphery	128 (69.2%)	67 (72.0%)	99 (72.8%)	64 (66.0%)	358 (70.1%)
	Total	185	93	136	97	511
Raccoon dog	Forest core	16 (76.2%)	35 (81.4%)	92 (80.0%)	110 (69.2%)	253(74.9%)
	Forest periphery	5 (23.8%)	8 (18.6%)	23 (20.0%)	49 (30.8%)	85 (25.1%)
	Total	21	43	115	159	338

### Fecal analysis

Fecal samples were collected and analyzed from 87 raccoons and 84 raccoon dogs from May to July in 2016 and 2017.

### Frequencies of food item occurrence

**Fig. 3:**
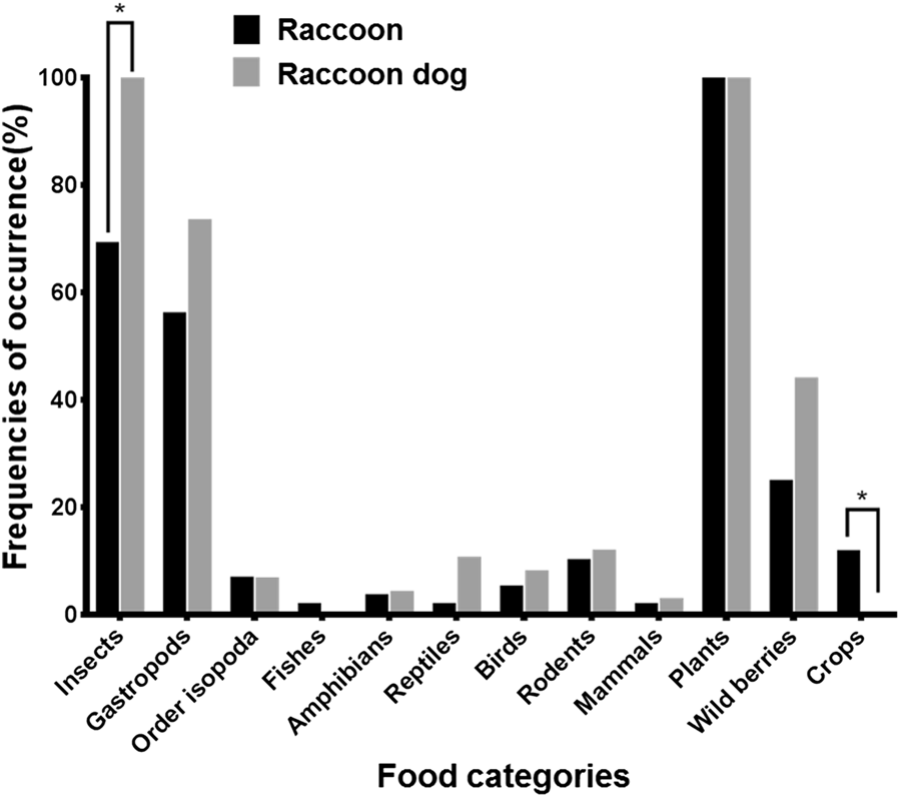
Frequency of occurrence (%) of each food category in raccoon and raccoon dog feces. *P < 0.004

For both raccoons and raccoon dogs, the top four food categories, in descending order, were: plants (herbaceous plants and woody forbs), insects, gastropods, and wild berries (Fig. 3). Plants were detected in all raccoon and raccoon dog feces (100%). Fisher’s exact test showed that the occurrence of insects was significantly higher in raccoon dog feces (P < 0.004); they were detected in all raccoon dog feces (100%). The occurrence of crops, mainly corn, was significantly higher in raccoon feces (11.5%, P < 0.004). Crops were not detected in raccoon dog feces (0%). Although gastropods (*Ezohelix gainesi* and *Succinea lauta*) and wild berries, which were all mulberry except for one sample (*Prunus sargentii*), occurred at higher frequencies in raccoon dog feces (gastropods, 72.7%; wild berries, 44.2%), there was no significant difference compared with raccoon feces (gastropods, P = 0.05; wild berries, P = 0.02). Other food categories were detected in the feces of both species at frequencies of occurrence less than 12%, with no significant differences between species (isopods, P = 1; mammals excluding wild rodents, P = 1; wild rodents, P = 0.789; birds, P = 0.731; reptiles, P = 0.077; amphibians, P = 1; and fish, P = 0.442).

### Food items consumed by percent volume

**Fig. 4:**
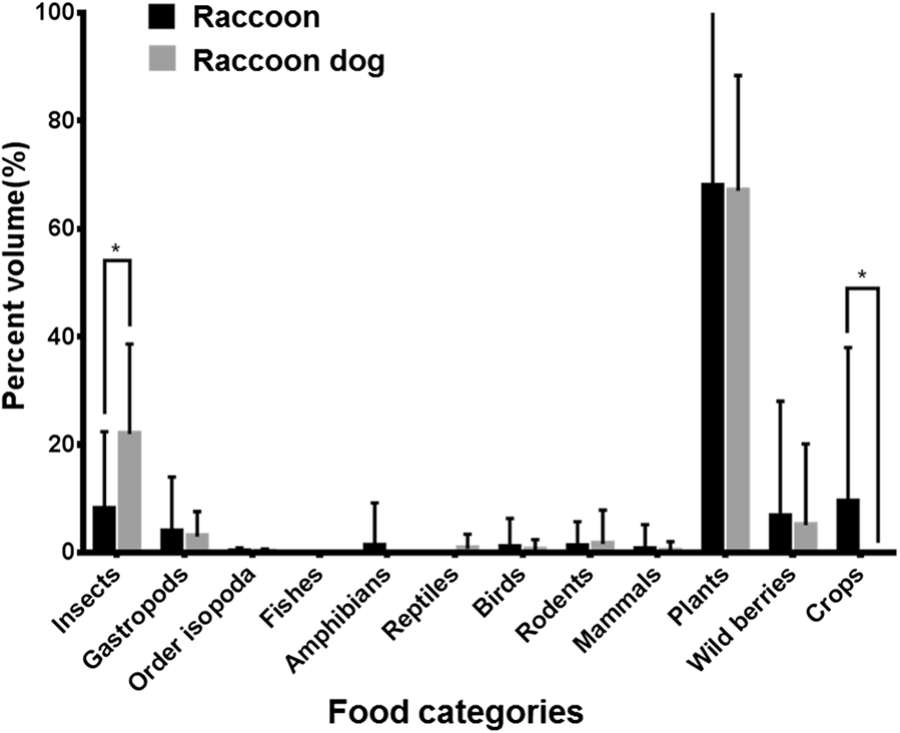
Percent volume (%) of each food category in raccoon and raccoon dog feces. *P < 0.004

For raccoons, the top four food categories, in descending order, were: plants, crops, insects, and wild berries (Fig. 4). For raccoon dogs, the top four food categories, in descending order, were: plants, insects, wild berries, and gastropods. A Mann–Whitney U test revealed that the percent volume of crops was significantly higher in raccoon feces (9.3%) than raccoon dog feces (P < 0.004), whereas the percent volume of insects was significantly higher (21.9%) in raccoon dog feces (P < 0.004). As with the frequency of occurrence, only insects and crops significantly differed. The percent volume of vertebrates, such as amphibians, was less than 2% in the feces of both species. The Shannon–Wiener diversity index for raccoons (0.43 ± 0.33, average ± SD) was significantly lower than that for raccoon dogs (0.69 ± 0.26) (Mann–Whitney U test, P < 0.05); the overlap index was 0.48 and significant (simulated overlap index with null model: 0.17, P < 0.05).

We identified insects, which are considered to be an important food resource for raccoon dogs, as much as possible in the feces of both raccoons and raccoon dogs, and calculated the frequencies of occurrence and the percent volume (Table 2). Terrestrial coleopterans, such as Carabidae species, were present at high levels in the feces of both species (frequencies of occurrence: raccoons, 69.0%; raccoon dogs, 100%). In particular, the frequency of occurrence of *Damaster blaptoides rugipennis* was high (raccoons, 34.5%; raccoon dogs, 60.6%). The next highest frequency of occurrence was *Terpnosia nigricosta* in the feces of both species (raccoons, 27.6%; raccoon dogs, 18.2%). *Vespula* spp. and *Bombus* spp. were found in raccoon feces (13.8% and 10.3%, respectively) but not in raccoon dog feces (0%).

**Table 2:** Frequency of occurrence (%) and percent volume of insects in raccoon and raccoon dog feces

Order	Family	Scientific name	Frequencies of occurrence(%)		Percent volume(%)	
			Raccoon	Raccoon dog	Raccoon	Raccoon dog
Coleoptera	Carabidae	*Damaster blaptoides rugipennis*	34.5	60.6	20.0	25.2
		*Damaster gehinii gehinii*	6.9	10.6	3.1	1.0
		*Carabus granulatus yezoensis*	3.5	18.2	0.0	1.6
		*Leptocarabus arboreus ishikarinus*	6.9	37.9	3.5	7.0
		*Leptocarabus opaculus opaculus*	6.9	12.1	0.0	0.6
		*Harpalidae* sp.	13.8	42.4	2.4	5.2
	Silphidae	*Silpha paerforata venatoria*	0.0	80.3	0.0	19.6
		*Dendroxena sexcarinata*	6.9	19.7	3.5	0.5
		*Nicrophorus quadripunctatus*	0.0	4.6	0.0	0.1
		*Nicrophorus maculifrons*	0.0	4.6	0.0	0.4
	Lucanidae	*Macrodorcas striatipennis*	6.9	28.8	2.3	2.3
		*Lucanus maculifemoratus*	0.0	19.7	0.0	5.7
		*Nipponodorcus rubrofemoratus*	0.0	1.5	0.0	0.5
	Geotrupidae	*Geotrupes laevistriatus*	3.5	40.9	2.3	6.8
	Scarabaeidae	*Copris ochus*	3.5	3.0	2.8	0.2
		*Onthophagus* sp.	0.0	1.5	0.0	0.2
		*Heptaphylla picea Motschulsky*	3.5	12.1	3.5	0.6
		*Eucetonia* spp.	3.5	4.6	0.0	0.6
	Erotylidae		0.0	3.0	0.0	0.0
	Curculionidae		0.0	4.6	0.0	0.2
	Dytiscidea		13.8	1.5	5.0	0.1
Hemiptera	Cicadidae	*Terpnosia nigricosta*	27.6	18.2	13.5	3.6
Orthoptera	Gryllotalpidae	*Gryllotalpa fossor*	6.9	4.6	1.2	0.1
Diptera	Calliphoridae		6.9	1.5	4.0	0.0
Hymenoptera	Formicidae		10.3	13.6	2.1	0.1
	Vespidae	*Vespula* spp.	13.8	0.0	5.6	0.0
	Apidae	*Bombus* spp.	10.3	0.0	4.0	0.0
Unidentified insects					21.4	18.0

### Stable isotope analysis

**Fig. 5:**
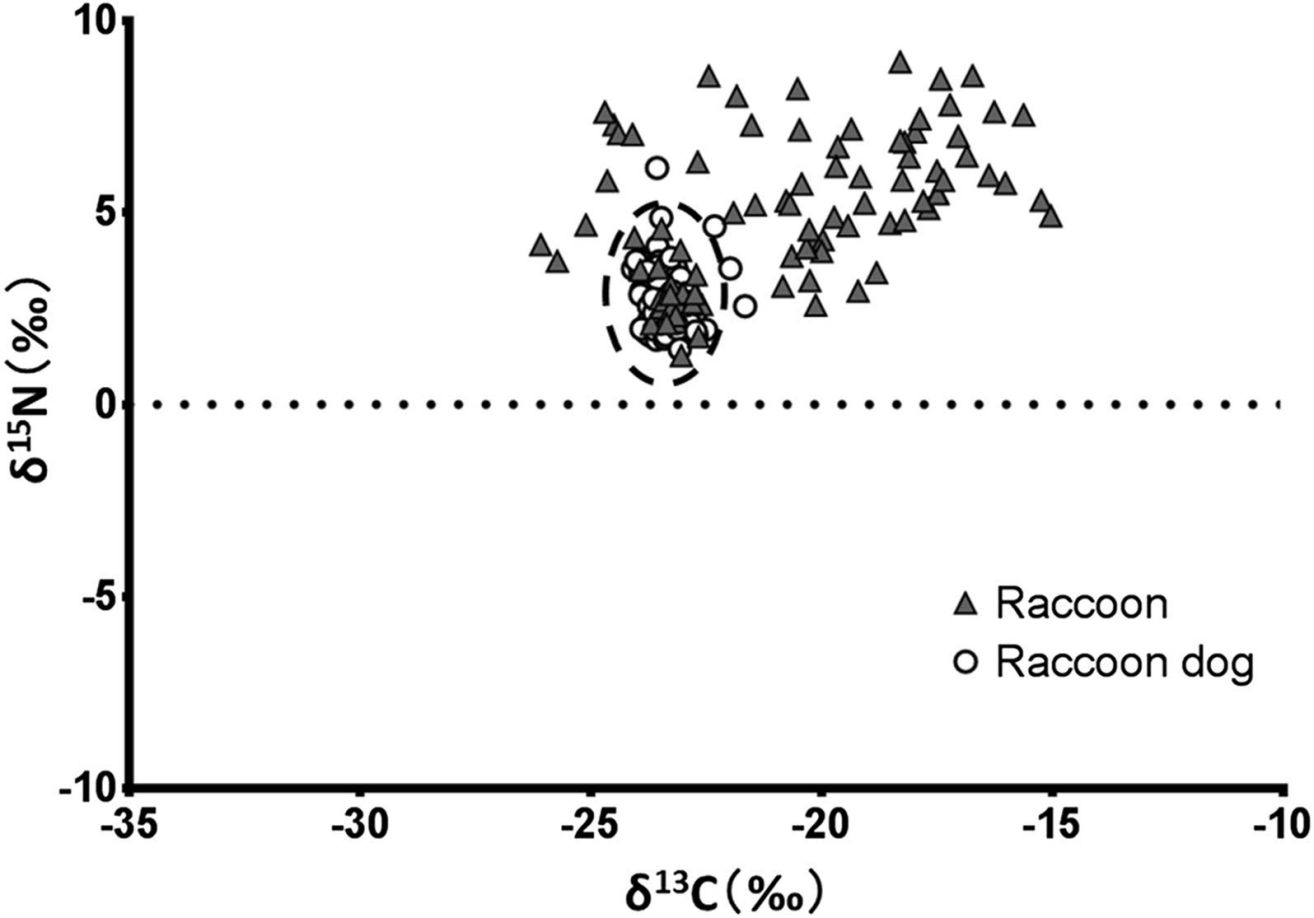
Stable isotope ratios of δ^13^C and δ^15^N in hair samples of raccoons and raccoon dogs. Values for raccoons are indicated by closed triangles, whereas those of raccoon dogs are indicated by open circles. The dotted line indicates stable isotope ratios that were similar between raccoons and raccoon dogs

**Fig. 6:**
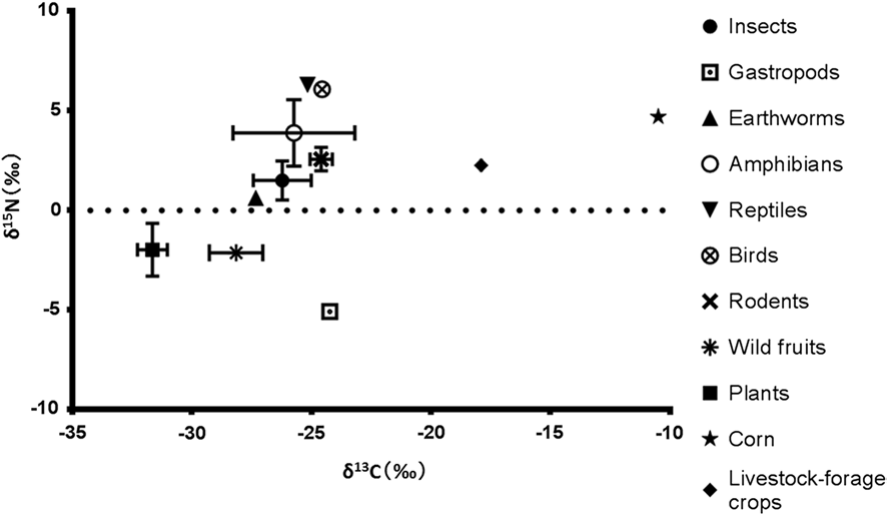
Stable isotope ratios of δ^13^C and δ^15^N for potential prey items of both species. Prey items were collected in Nopporo Natural Forest Park between 2016 and 2017. Symbols indicate mean ± SE

We collected hair samples from 93 raccoons and 86 raccoon dogs between 2016 and 2017. Stable isotope results of hair and potential prey items of both species are shown in Figs. 5, 6, Tables 3, 4 and Additional file 1. The δ^13^C and δ^15^N values for raccoons were − 20.82‰ ± 2.76‰ (average ± SD) and 4.85‰ ± 1.97‰, respectively; those for raccoon dogs were −23.27‰ ± 0.48‰ and 2.72‰ ± 0.78‰, respectively. Standard deviations of the isotope analysis were within 0.2‰ for both δ^13^C and δ^15^N. Both δ^13^C and δ^15^N values were high and had greater ranges in raccoons than raccoon dogs. Both δ^13^C and δ^15^N values of raccoons varied widely, whereas those of raccoon dogs were low, and individual differences were also small (Fig. 5).

**Table 3:** Stable isotope results of raccoon hair

No.	δ^13^C	δ^15^N	No.	δ^13^C	δ^15^N
1	− 23.4	2.82	51	− 24.1	4.34
2	− 23.5	2.57	52	− 18.3	8.92
3	− 23.1	2.43	53	− 17.5	6.08
4	− 23.2	2.53	54	− 22.4	8.55
5	− 20.2	4.23	55	− 20.5	8.22
6	− 26.1	4.16	56	− 18.5	4.71
7	− 23.4	2.12	57	− 16.9	6.47
8	− 23.1	4.01	58	− 17.5	5.48
9	− 21.9	4.99	59	− 23.4	2.70
10	− 23.7	2.09	60	− 22.7	2.69
11	− 23.0	2.74	61	− 20.1	2.58
12	− 19.7	4.87	62	− 18.2	4.79
13	− 23.5	2.74	63	− 19.7	6.20
14	− 25.1	4.67	64	− 23.0	2.89
15	− 23.5	4.57	65	− 17.8	5.29
16	− 24.5	7.28	66	− 22.6	2.60
17	− 24.7	7.61	67	− 18.3	6.86
18	− 20.6	3.86	68	− 23.3	2.48
19	− 20.8	5.30	69	− 24.4	7.05
20	− 17.7	5.10	70	− 23.2	2.31
21	− 20.4	5.74	71	− 16.4	5.96
22	− 15.6	7.54	72	− 18.8	3.43
23	− 18.0	7.10	73	− 23.3	2.86
24	− 17.2	7.80	74	− 19.2	2.95
25	− 23.9	3.49	75	− 22.8	2.64
26	− 16.7	8.56	76	− 16.0	5.76
27	− 21.8	8.03	77	− 20.3	3.22
28	− 23.3	3.10	78	− 24.1	7.03
29	− 17.9	7.43	79	− 17.4	5.83
30	− 24.6	5.82	80	− 20.3	4.08
31	− 23.5	2.51	81	− 15.2	5.31
32	− 23.5	3.55	82	− 21.5	7.27
33	− 19.2	5.92	83	− 15.0	4.91
34	− 20.5	7.15	84	− 22.7	1.76
35	− 18.2	6.83	85	− 22.7	2.88
36	− 19.7	6.71	86	− 23.4	2.10
37	− 17.0	6.98	87	− 23.0	1.27
38	− 20.7	5.21	88	− 20.8	3.07
39	− 21.4	5.19	89	− 20.3	4.54
40	− 18.1	6.44	90	− 19.4	4.65
41	− 17.5	5.51	91	− 22.7	3.37
42	− 18.2	5.84	92	− 16.3	7.62
43	− 20.0	4.29	93	− 17.4	8.47
44	− 20.0	3.98			
45	− 19.1	5.24			
46	− 23.2	2.14			
47	− 19.4	7.17			
48	− 23.1	2.57			
49	− 22.7	6.31			
50	− 25.7	3.73			
			Average	− 20.82	4.85
			SD	2.76	1.97

**Table 4:** Stable isotope results of raccoon dog hair

No.	δ^13^C	δ^15^N	No.	δ^13^C	δ^15^N
1	− 23.5	3.01	51	− 23.6	2.38
2	− 23.8	3.33	52	− 22.8	1.81
3	− 23.6	4.12	53	− 23.4	2.40
4	− 23.7	2.89	54	− 23.2	2.26
5	− 23.0	2.37	55	− 22.9	2.22
6	− 23.5	2.74	56	− 23.3	2.86
7	− 23.2	3.54	57	− 22.8	2.72
8	− 23.2	2.25	58	− 22.9	2.33
9	− 23.9	3.75	59	− 22.9	2.45
10	− 23.7	1.83	60	− 21.7	2.56
11	− 23.1	2.98	61	− 23.4	1.80
12	− 23.8	1.87	62	− 23.1	2.71
13	− 22.0	3.54	63	− 23.1	1.96
14	− 23.5	3.73	64	− 23.5	4.86
15	− 23.4	2.31	65	− 23.0	2.35
16	− 23.6	3.12	66	− 23.1	3.31
17	− 23.7	1.79	67	− 23.0	2.26
18	− 24.1	3.53	68	− 23.2	2.95
19	− 23.2	2.70	69	− 23.6	2.76
20	− 23.8	3.31	70	-22.8	2.31
21	− 23.2	2.55	71	− 22.8	2.16
22	− 23.8	2.93	72	− 23.1	2.13
23	− 23.9	2.96	73	− 23.1	2.48
24	− 23.7	2.70	74	− 23.0	2.41
25	− 23.5	3.61	75	− 23.3	2.32
26	− 23.9	3.43	76	− 22.3	4.64
27	− 23.6	1.68	77	− 22.7	2.42
28	− 23.3	3.82	78	− 23.1	2.66
29	− 23.4	1.63	79	− 23.0	2.53
30	− 23.7	2.73	80	-23.1	2.55
31	− 23.9	2.86	81	− 22.9	2.91
32	− 23.8	3.11	82	− 22.9	2.68
33	− 23.4	2.71	83	− 22.9	2.12
34	− 23.7	3.68	84	− 22.8	2.65
35	− 23.5	3.25	85	− 23.1	1.44
36	− 21.9	3.55	86	− 22.7	1.90
37	− 24.0	3.74			
38	− 23.7	2.20			
39	− 23.9	1.97			
40	− 23.8	2.56			
41	− 23.8	3.48			
42	− 23.6	1.95			
43	− 23.0	2.47			
44	− 23.6	2.48			
45	− 23.6	6.16			
46	− 23.4	1.71			
47	− 22.5	1.95			
48	− 23.0	2.06			
49	− 23.1	2.44			
50	− 22.7	1.79			
			Average	− 23.27	2.72
			SD	0.48	0.78

**Fig. 7:**
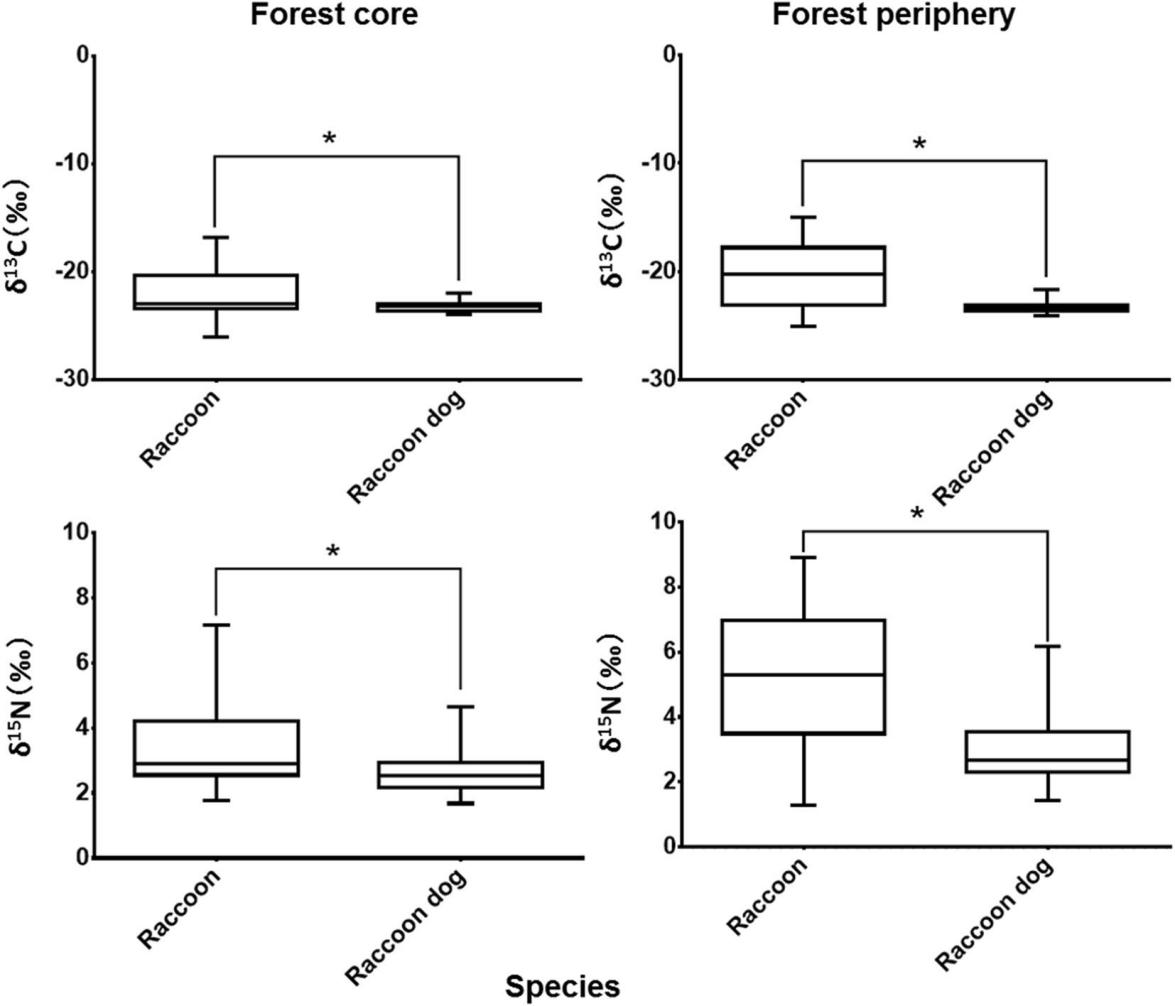
Comparison of δ^13^C and δ^15^N stable isotope ratios in hair samples from both species. Left side, the forest core; right side, forest periphery. *P < 0.01

A three-way ANOVA revealed significant interaction effects between species and capture sites for both δ^13^C and δ^15^N (δ^13^C: F [1, 167] = 3.883, MSe = 3.824, P < 0.05; δ^15^N: F [1, 167] = 8.863, MSe = 1.896, P < 0.05). No significant main effects were found for hair type (summer and winter coats) (δ^13^C: F [1, 167] = 0.039, MSe = 3.824, P = 0.844; δ^15^N: F [1, 167] = 0.618, MSe = 1.896, P = 0.433), and interaction effects between each factor for both δ^13^C and δ^15^N (δ^13^C: hair type × species, F [1, 167] = 0.268, MSe = 3.824, P = 0.606; hair type × capture sites, F [1, 167] = 0.00, MSe = 3.824, P = 0.995; hair type × species × capture sites, F [1, 167] = 0.024, MSe = 3.824, P = 0.877; δ^15^N: hair type × species, F [1, 167] = 2.196, MSe = 1.896, P = 0.140; hair type × capture sites, F [1, 167] = 1.344, MSe = 1.896, P = 0.248; and hair type × species × capture sites, F [1, 167] = 0.56, MSe = 1.896, P = 0.455). Bonferroni analysis showed that, for capture sites, δ^13^C and δ^15^N in both the forest periphery and the forest core were significantly higher in raccoons than raccoon dogs (forest core: δ^13^C and δ^15^N, P < 0.01; forest periphery: δ^13^C and δ^15^N, P < 0.01) (Fig. 7). Additionally, δ^13^C and δ^15^N in raccoon hair were significantly higher in the forest periphery than the forest core (δ^13^C and δ^15^N: P < 0.01).

We found that 31.2% (29/93) of raccoons had stable isotope ratios similar to those of raccoon dogs (the similarities are indicated within the dotted line in Fig. 5). Age assessment of these 29 raccoons using their skulls revealed that 15 were juvenile raccoons, 11 were yearlings, and three were over 2 years old (Table 5). Thus, 89.7% (26/29) of the raccoons were less than 2 years old, and 75.9% (22/29) were captured in the forest core and the forest periphery near the urban area (A, B, C), which was inhabited by many raccoon dogs. Although the amount of farmland was not large, there were many wild fruits and insects at points A, B, and C.

**Table 5:** Age structure of raccoons with stable isotope ratios similar to those of raccoon dogs

Capture site	Forest core			Point A, B, C				Forest periphery			
Age	0	1	≥ 2	0	1	≥ 2	Total	0	1	≥ 2	Total
Male	6	3	0	0	2	0	11	1	0	0	1
Female	4	1	1	1	3	1	11	3	2	1	6
Total	10	4	1	1	5	1	22	4	2	1	7

We conducted an age assessment of these 29 raccoons using their skulls

## Discussion

Fecal analysis is commonly used to investigate the feeding habits of wildlife. In Japan, most feeding habit analyses of medium-sized mammals use fecal or stomach contents of wildlife that were captured by nuisance control. However, because wildlife are captured near human houses, fecal or stomach contents often include human garbage [22]. Therefore, it is difficult to accurately determine the feeding habits of these animals. Because capture sites often differ among animals captured by nuisance control, it is challenging to conduct accurate comparisons of invasive and native species [23]. In the current study, we investigated raccoon and raccoon dog feeding habits and habitat use in an isolated forest to more accurately infer feeding habits and habitat use.

Fecal analysis revealed that raccoons and raccoon dogs are opportunistic omnivores; they consume easily found food items, such as plants, insects, gastropods, and wild berries, all of which occurred in high levels in their feces. However, the occurrence of crops, mainly corn, was significantly higher in raccoon feces, whereas the occurrence of insects was significantly higher in raccoon dog feces.

The Shannon–Wiener diversity index for raccoons was significantly lower than that for raccoon dogs. A higher Shannon–Wiener diversity index indicates that food diversity is higher and that the proportion of food consumed from each food category is nearly equal [24]. Raccoons are more selective in what they consume than raccoon dogs, which may have resulted in bias of food items chosen by raccoons. We suggest that raccoons tend to be most attracted to highly palatable foods and might be more prone to moving into human-inhabited areas to eat crops, even though there are abundant food resources in the forest. This finding is consistent with the results of previous research in North America, which found that raccoons prefer corn to naturally available foods because they find it highly palatable [25–27]. Conversely, raccoon dogs appear not to be as driven by food palatability as raccoons, and raccoon dogs appeared to prefer to eat foods available in the forest. However, raccoon dogs also consume food items of relatively high palatability, such as insects. Sako et al. (2008) reported the frequency of occurrence of insects and wild fruits, including wild berries, in raccoon dog feces to be approximately 90% [28]. Although our results regarding insects (100% occurrence) are similar, the frequency of occurrence we found for wild berries (44.2%) was lower (Fig. 3), probably because wild berries did not bear fruit in our study area during the study period—the wild berries that did occur in feces were Aino mulberry (*Morus australis*), which bears fruit in July. Moreover, wild fruits like hardy kiwi (*Actinidia arguta*) and crimson glory vine (*Vitis coignetiae*) occurred at high levels in raccoon dog latrines during autumn (Sashika M., personal observation). Therefore, raccoon dogs likely eat wild fruits that are highly palatable.

A palatability test is needed to adequately determine food palatability; however, this is difficult to perform in the wild because other non-target wildlife often eat prepared food. Moreover, research conducted using captive animals in zoo environments is confounded by the preference shown by animals to eat food to which they have become accustomed (data not shown). Therefore, it is very difficult to determine wildlife food palatability. However, it appears that raccoons had a greater preference for corn than raccoon dogs. It was difficult to capture raccoons—even when traps were placed around cornfields—when the corn bore fruit, because raccoons did not enter the traps but instead damaged the cornfield. Corn was not detected in the feces of raccoon dogs caught near a cornfield.

The overlap index showed that the food resources of raccoons and raccoon dogs overlapped (overlap index: 0.48, simulated overlap index with null model: 0.17, P < 0.05). However, the relative contributions of these categories to total diet differed between species. This result indicates that raccoons and raccoon dogs ate the same food categories, but the food items consumed differed. Additionally, highly digestible foods were probably underestimated and needed to be analyzed based on stable isotope results.

The differences in food items consumed between these species are likely related to habitat selection. There is a close relationship between feeding behavior and habitat selection. Wildlife select habitats where they can more easily find necessary food resources [29]. When we started this research in 2004, the number of captured raccoons and raccoon dogs was less than half of those captured at the time of writing. At that time, there was enough space for both species in the forest. So, we can surmise that if raccoon dogs preferred the forest periphery, they would have moved to forest periphery; and if raccoons preferred the forest core, they would have inhabited the forest core. However, most raccoons were captured at the forest periphery close to agricultural land (70.1%), where they can more easily access agricultural land and there are greater opportunities to consume crops. These results are similar to those of Abe (2006) [6]. Conversely, most raccoon dogs were captured in the forest core (74.9%), which is where there are more opportunities to eat insects and wild fruits. Since the raccoon dog population began recovering in 2014, the number of captured raccoon dogs at the forest periphery (especially trapping points A, B, and C, where there were many wild fruits and insects) has gradually increased. It is possible that the raccoon dogs moved to the forest periphery in search of food resources because space in the forest core decreased as the raccoon dog population recovered. Therefore, they might have moved to areas where they could easily obtain preferred food items.

Although we did not analyze trophic discrimination factors because of animal welfare concerns, we observed differences between raccoons and raccoon dogs. The δ^13^C and δ^15^N ratios in raccoon hair were significantly higher than those in raccoon dog hair. Both the δ^13^C and δ^15^N values for raccoons were also larger. We speculate that raccoons had a high range of δ^13^C values because they consumed corn and livestock-forage crops, which had the highest δ^13^C values of potential food items in our study area because they include C4 plants. When large amounts of crops and human garbage are consumed by wildlife, the δ^13^C values are over − 21‰ [30]. In this study, the percentage of raccoons with δ^13^C above − 21‰ was 50.5% (47/93). Furthermore, δ^13^C values for raccoon hair were significantly higher for raccoons captured at the forest periphery than for raccoons captured in the forest core. These results indicate that many raccoons frequently visited the farmland outside Nopporo Natural Forest Park, which is consistent with reports of corn damage by farmers neighboring our study area (Sashika M., personal observation).

Reptiles, amphibians, and corn showed high δ^15^N values, which indicated that raccoons may be more likely to eat them than raccoon dogs. However, the incidence of amphibian and reptile remains in raccoon feces was low. Additionally, although raccoons may prey on Hokkaido salamanders (*Hynobius retardatus*), of which the local population in Hokkaido is threatened, as suggested by leftover remains of Hokkaido salamanders (Abe G., personal observation), we are unaware of any report of salamanders found in raccoon stomach contents or feces. We speculate that these highly digestible foods were underestimated in our fecal analysis. Detection rate varies depending on digestibility; for example, plant matter with low digestibility is more likely to be found in feces and result in underestimation of animal matter consumption [31]. Moreover, the δ^15^N values from raccoon hair were significantly higher for raccoons captured at the forest periphery than for raccoons captured in the forest core. Salamanders, frogs, and snakes, which all have high δ^15^N values, are mainly located in the forest. However, some salamanders, frogs, and snakes also occur in rice fields and agricultural ponds near the forest. Therefore, their distribution is nearly the same in the forest periphery and the forest core. Consequently, the most likely causes of high δ^15^N values are corn, human leftovers, and garbage.

Results of our fecal and stable isotope analyses demonstrated that raccoons depend on corn as a food resource, although the highest δ^15^N value, 8.92, was reported for a raccoon, which indicates that high δ^15^N values were not entirely from corn. The δ^15^N value of human hair in Japan exceeds 10.0‰, which indicates that leftover human food also has similarly high δ^15^N values [31]. Raccoons captured in the forest periphery may have been eating corn and highly digestible garbage from the private houses that surround the forest.

Both δ^13^C and δ^15^N values were low in raccoon dog hair, and individual differences were also small. The δ^13^C and δ^15^N values of raccoon dog hair are similar to values for common forest resources such as insects [32], wild fruits, and earthworms, on which raccoon dogs probably feed. This result is consistent with results from our fecal analysis. Because no differences in δ^13^C and δ^15^N values were found between raccoon dogs captured in different locations, it is possible that raccoon dogs rarely leave the forest to feed, which is a finding that is consistent with previous research on the home ranges of raccoon dogs at Nopporo Natural Forest Park [6].

It is possible that raccoons and raccoon dogs with overlapping stable isotope ratios had similar feeding habits, because fecal analysis revealed that raccoons also ate insects and wild fruits. Of the insects observed, terrestrial coleopterans and *T*. *nigricosta* were present at high levels in the feces of both species. The percent volume of *T. nigricosta* was low relative to coleopterans. This could be because the exoskeleton of *T. nigricosta* is light. During the hatching period, *T. nigricosta* is easy to eat. *T. nigricosta* is also considered an important food resource for raccoons and raccoon dogs. Raccoons with ratios that overlapped with those of raccoon dogs were captured in the same locations where many raccoon dogs had been captured. Insects are widely distributed in the forest, as are many plant species that produce wild fruits (such as Aino mulberry and hardy kiwi). Although our findings provide indirect evidence of dietary preference, it was still apparent that the feeding habits and habitat use of these raccoons overlapped with those of raccoon dogs. Most (about 90%) of the raccoons with stable isotope ratios that overlapped with those of raccoon dogs were younger than 2 years old, and they were the minority (31.2%) of raccoons captured.

We then evaluated whether the habitat and food resources of raccoons and raccoon dogs overlapped. It is possible that if there were enough forest space, habitat and food resources would not overlap. After the raccoon dog population recovered, there was probably not sufficient space in the forest, and raccoon dogs likely moved to the forest periphery. If raccoons had not been imported into Japan, raccoon dogs would likely remain in their preferred habitat. However, now that there are raccoons in the forest, the habitat and food resources of raccoon dogs appear to overlap with those of raccoons. The raccoon population in the study area is maintained at a low density by the feral raccoon control program. This may be limiting overlapping habitat use and feeding habits between the two species. However, if the population density of raccoons increases, their feeding habits and habitat use may begin to overlap more with those of raccoon dogs. Because the feral raccoon control program limits the density of raccoons within the forest, the current feeding habits and habitat use of raccoons do not appear to overlap sufficiently with those of raccoon dogs to impact them. Thus, if raccoon numbers remain low, the impact on raccoon dogs will be limited. However, even at observed low population sizes, the feeding habits and habitat use of some raccoons did overlap somewhat with those of raccoon dogs. Should the density of raccoons increase, raccoons could negatively affect the native raccoon dog population. Our results suggest that the feral raccoon control program is likely protecting native raccoon dogs. Therefore, we advocate continuing this program.

Our study provides relatively accurate data on raccoon and raccoon dog, which is otherwise difficult to obtain, because it was conducted in an isolated forest. In addition, although Japanese raccoon dogs are endemic to Japan, they are canids, so our research can also be applied to future research on other canids worldwide, such as red foxes. Additionally, raccoon dogs from the Eastern Asia continental population have been introduced into European countries [33]; therefore, our results may be useful in identifying potential competition between raccoon dogs and native canids in such environments. Moreover, stable isotope analysis of medium-sized mammals has not progressed greatly [34]. Therefore, we believe this research will help inform future studies on medium-sized mammals, particularly canids.

## Conclusions

We demonstrated that raccoons and raccoon dogs are typical opportunistic omnivores that consume easily found food items. Although we provided indirect evidence of food preference, both species clearly consumed different food resources, probably because of differences in food preference. The preferred habitats of each species also differed, with each species tending to be found in areas in which it could easily obtain its preferred foods. If raccoon density remains low, the feeding habits and habitat use of these two species will likely not sufficiently overlap to negatively impact the native raccoon dog population. However, because some overlap between feeding habits and habitat use occurs, it is important to continue the raccoon control program so that the impact of raccoons does not increase.

## Methods

### Study area and period

This study was conducted in Nopporo Natural Forest Park, 11–15 km east of Sapporo City, central Hokkaido, Japan (43° 25′ N, 141° 32′ E) (Fig. 1). Nopporo Natural Forest Park is a semi-isolated forest with an area of 2053 ha and an altitude of 20–100 m above sea level, and it is surrounded by residential areas and farmland. The subarctic climate has a mean temperature of 7.4 °C (− 21.7 °C in January to 32.7 °C in July) and mean annual precipitation of 1064 mm (data from the Japan Meteorological Agency). The forest is mixed conifer–hardwood and dominated by Sakhalin fir (*Abies sachalinensis*), painted maple (*Acer pictum* subsp. *Mono*), water oak (*Quercus mongolica* var. *grosseserratus*), and Manchurian ash (*Fraxinus mandshurica* var. *japonica*) [35]. Despite being located near Sapporo City, rich and diverse environments remain in Nopporo Natural Forest Park; 21 mammal (including raccoon dogs, red foxes, and martens), 150 bird, and 1300 insect species are known to inhabit this area. Raccoons were first confirmed in this park in 1992 [36].

Because of agricultural damage caused by raccoons, the Hokkaido Government initiated a feral raccoon control program in Nopporo Natural Forest Park in 1999. Since then, the raccoon population has been maintained at low densities [7]. Because this forest provides an environment preferred by raccoons, when population densities are low, new raccoons invade the forest from surrounding areas [7]. The forest’s raccoon population has decreased to less than 2 individuals/km^2^ since 2014 (Hokkaido Prefecture, unpublished data).

Raccoons and raccoon dogs have been captured in Nopporo Natural Forest Park since 2004. Surveys were conducted from May to July; when food resources for raccoons and raccoon dogs increase in autumn, there is less scarcity and they do not need to eat the food in the traps; therefore, they are less likely to enter the traps [7].

### Sample and data collection for habitat use and feeding habit analyses

Raccoons and raccoon dogs were captured in box traps (Havahart Large Collapsible Pro Cage Model 1089, Woodstream Corp., Lititz, PA, USA) baited with dog food, corn snacks, and doughnuts. Traps were placed at 80 to 100 sites. Because of natural disasters, such as typhoons, places where we could place traps differed from year to year. The traps were placed in a square grid at 500-m intervals. The interval size between the traps was based on the raccoon minimum home range reported in Hokkaido (25.5 ha) and should have covered at least a part of the home range of all raccoons inhabiting the survey area [37]. Trapping points within 250 m of the forest boundary line were defined as forest periphery sites (41 points), and other points in the forest were considered the forest core (59 points), as described by Abe (2008) [7]. A Chi squared test was performed to compare the number of raccoons and raccoon dogs captured at the two regions (forest periphery vs. the forest core). Differences were considered statistically significant at P< 0.05.

Captured raccoon dogs were anesthetized with butorphanol tartrate (Vetorphale 5 mg, 1.2 mg/kg; Meiji Seika, Tokyo, Japan), hydrochloric acid medetomidine (Dolbene, 40 µg/kg; Kyoritsu, Tokyo, Japan), and midazolam (Dormicum injection 10 mg, 0.2 mg/kg; Astellas, Tokyo, Japan) by intramuscular injection before handling. Microchips were subdermally inserted into their backs to aid identification of individual animals. Body size and sex of each raccoon dog were recorded, and hair samples were collected from the back of each individual along with fecal samples found within traps. Hair samples were wrapped in paper and stored in plastic bags. After sampling and measurement, we administered the antagonists naloxone (Naloxone hydrochloride intravenous injection, 0.02 mg/kg; DAIICHI SANKYO, Tokyo, Japan), atipamezole hydrochloride (Atipame, 0.2 mg/kg; Kyoritsu, Tokyo, Japan), and flumazenil (Flumazenil intravenous injection 0.5 mg, 0.02 mg/kg; Sawai, Osaka, Japan). The raccoon dogs were released at the capture site after they fully recovered from the anesthesia. For any raccoon dog recaptured within the same month, we merely scanned their microchips and released them. For individuals recaptured in different months, fecal and hair samples from individuals were collected and considered separate samples.

Captured raccoons were first anesthetized similar to raccoon dogs and then euthanized by injection with potassium chloride into the heart. Carcasses were collected as part of the feral raccoon control program. Raccoon body size and sex were recorded, hair samples from their back and fecal samples from their rectum were collected, and their skulls were taken to estimate age. Hair samples from raccoons captured for nuisance control and a raccoon dog carcass found in Nopporo Natural Forest Park were also collected. Potential food items (such as plants, wild fruits, and insects using pitfall traps) were collected in the forest (Table 6). Samples were stored at − 20 °C until assays were performed.

**Table 6:** Potential prey items

Prey item	Taxon
Plant material	
Herbaceous plants	*Sasa* spp.
	*Quercus* spp.
	*Acer* spp.
Wild fruits	*Morus bombycis*
	*Actinidia arguta*
	*Vitis coignetiae*
	*Castanea crenata*
Crops	*Zea mays*
Other	Livestock-forage crops
Animal materials	
Insects	*Damaster blaptoides rugipennis*
	*Carabus granulatus yezoensis*
	*Leptocarabus arboreus ishikarinus*
	*Anisodactylus*
	*Silpha paerforata venatoria*
	*Eusilpha japonica*
	*Plesiophthalmus nigrocyaneus nigrocyaneus*
	*Vespula austriaca*
	*Terpnosia nigricosta*
Gastropoda	*Ezohelix gainesi*
Order Isopoda	*Oligochaeta* spp.
Amphibia	*Hynobius retardatus*
	*Rana pirica*
	*Hyla japonica*
	*Glandirana rugosa*
	*Pelophylax nigromaculatus*
Reptilia	*Elaphe climacophora*
Aves	*Hypsipetes amaurotis*
Mammalia	*Apodemus speciosus ainu*
	*Apodemus argenteus*

### Fecal analysis

For fecal analysis, we examined the samples collected between 2016 and 2017. We used the point-frame method to quantitatively evaluate the composition of food eaten by individual animals. The amount of each food type within feces was quantified by surface area. The point-frame method is a straightforward way to calculate the frequency of food in feces, and has been used for various animal species, such as ungulates and carnivores [38]. In this study, fecal samples were sterilized overnight at 70 °C to kill *Echinococcus multilocularis* and then washed with tap water over a 0.5-mm mesh sieve; then, the material that remained on the sieve was spread onto a laboratory dish (10-cm diameter). The bottom of the dish was marked with a 5 × 5-mm grid, and the intersection points of the grid lines were the point frames. We identified each food item that touched an intersection point and counted the number of points each item touched. Samples that touched more than 200 points were saved to analyze frequency of occurrence (%), percent volume, and overlap index of each food category [38]. Food items were placed into 12 categories: wild berries and seeds, plants (herbaceous plants and woody forbs), crops, insects, gastropods, isopods, mammals (except wild rodents), wild rodents, birds, reptiles, amphibians, and fish. We collected potential food items (such as wild fruits, plants, and insects) in this forest, and we received corn and livestock-forage crops from farmers near the forest. When we conducted fecal analysis, we compared features of the food items detected from the feces of raccoons and raccoon dogs with collected food samples. Then, we classified the food items into the 12 categories. Plants, wild fruits, and crops tend to have low digestibility, and their epidermis and seeds often remain visible in raccoon and raccoon dog feces. Therefore, it was possible to distinguish plants, wild fruits, and crops. Groomed hair, artificial materials, stones, and gravel were excluded from analysis because they had likely been unintentionally eaten. Individual food items were identified and recorded. We calculated the frequency of occurrence (%) and percent volume of each food category. Fisher’s exact test was used to compare the frequency of occurrence, and a Mann–Whitney U test was used to compare the percent volume of each category between species. Both tests were corrected by the Bonferroni method. Differences were considered statistically significant at P < 0.004.

Based on the proportion of food items, we calculated the diversity of food items and overlap of food items consumed by raccoons and raccoon dogs. Food diversity was evaluated using the Shannon–Wiener diversity index formula [24]:

$$Hi = - \sum {\left( {P_{i} } \right)} \ln \left( {P_{i} } \right)$$

where *P*_*i*_ is the proportion of food item *i* in the total diet. A higher *Hi* value indicated greater food diversity.

The overlap of foods between the two species was calculated with the Pianka index (a_jk_) formula [39]:

$${\text{a}}_{\text{jk}} = \sum {\left( {P_{ij} } \right)} \left( {P_{ik} } \right)/\left[ {\left( {\sum {P_{ij} } } \right)^{2} \left( {\sum {P_{ik} } } \right)^{2} } \right]^{1/2}$$

where *P*_*ij*_ and *P*_*ik*_ are the proportions of food item *i* in the total diet of species *j* and *k*, respectively. When the overlap index value a_jk_ is equal to zero, food items consumed by the two species, *j* and *k*, do not overlap at all; when a_jk_ is 1, consumed food items overlap completely. Accordingly, 0 ≤ a_jk_ ≤ 1. Pianka’s index was used to test significance using the RA3 algorithm in EcoSim 7.72 to compare the observed overlap values and the distribution of expected overlap values based on a null model that was generated by 1000 repetitions [40, 41]. If the observed values were higher or lower than 95% of the simulated indices, they were considered statistically different from the null distribution values. A significantly lower observed value indicates differences in diet or food resource partitioning, whereas a significantly higher observed value indicates similar diets or strong resource competition [40, 41].

A Mann–Whitney U test was performed to compare the Shannon–Wiener diversity index between the two species, and differences were considered statistically significant at P < 0.05.

### Stable isotope analysis

Samples of potential prey items were dried at 60 °C for over 24 h, and then ground with a mortar and pestle. Potential prey items and hair samples were rinsed with a 2:1 chloroform:methanol solution to remove lipids and then dried at 60 °C for at least another 24 h [31]. Hair from each captured animal was analyzed to generate average hair values for both species. Samples were enclosed in a tin cup and combusted in an elemental analyzer (vario MICRO cube, Elementar Gmbh, Langersell Bolt, Germany) interfaced with an isotope ratio mass spectrometer (IsoPrime100, Elementar Gmbh, Langersell Bolt, Germany).

We determined the carbon (δ^13^C) and nitrogen isotope (δ^15^N) ratios for each sample. Results are reported as parts per thousand of the isotope relative to a standard. We used the Pee Dee Belemnite standard for carbon, and atmospheric nitrogen as the standard for nitrogen [31]. The assessment of feeding ecology of the two species from stable isotope ratios was based on the trophic discrimination factors described by previous studies (δ^15^N: 3.4 ‰, δ^13^C: 2.6 ‰) [16–18, 42]. We also considered that both raccoons and raccoon dogs are omnivorous and that this analysis reveals average values for hair. We did not conduct experiments on the trophic discrimination factors of both species in this study because we thought that continuing to give only one kind of food to omnivorous species for several months is contrary to animal welfare.

We performed three-way ANOVAs to examine interactions between three independent variables, species (raccoon vs. raccoon dog), hair type (summer coat vs. winter coat), and capture site (forest periphery vs. the forest core), on the stable isotope ratio dependent variables (δ^13^C and δ^15^N). After we examined interactions among three independent variables (species, capture site, and hair type), a Mann–Whitney U test was conducted to compare the between independent variables. Then, the tests were corrected by the Bonferroni method. Differences were considered statistically significant at P < 0.01.

GraphPad Prism6 (GraphPad Software, San Diego, CA, USA) and SPSS Statistics 20.0 (IBM, Tokyo, Japan) were used for all statistical analyses.

Information on the process of hair growth is included in Additional file 2.

## Supplementary information

**Additional file 1.** Stable isotope ratios of δ^13^C and δ^15^N for potential prey items.

**Additional file 2.** Information on the process of hair growth.

## Data Availability

The datasets used and/or analysed during the current study are available from the corresponding author on reasonable request.

## Abbreviations

a_jk_: Pianka index
*H*: Shannon–Wiener diversity index
*P*_*i*_: the proportion of food item *i* in the total diet
*P*_*ij*_: the proportions of food item *i* in the total diet of species *j*
*P*_*ik*_: the proportions of food item *i* in the total diet of species *k*
δ^13^C: carbon stable isotope
δ^15^N: nitrogen stable isotope

## References

[1] Gehrt S, Feldhamer G, Thompson B, Chapman J (2003). Raccoons and allies. Wild mammals of North America.

[2] Ikeda T, Asano M, Matoba Y, Abe G (2004). Present status of invasive Alien Raccoon and its impact in Japan. Glob Environ Res..

[3] Ikeda T, Takatsuki S, Yamagiwa J (2008). Invasive alien species issues, with special reference to raccoons. Mammalogy in Japan.

[4] Sashika M, Abe G, Matsumoto K, Inokuma H (2010). Molecular survey of rickettsial agents in feral raccoons (*Procyon lotor*) in Hokkaido, Japan. Jpn J Infect Dis..

[5] Yamaguchi E, Sashika M, Fujii K, Kobayashi K, Bui VN, Ogawa H, Imai K (2014). Prevalence of multiple subtypes of influenza A virus in Japanese wild raccoons. Virus Res.

[6] Abe G, Ikeda T, Tatsuzawa S. Differences in habitat use of the native raccoon dog (*Nyctereutes procyonoides albus*) and the invasive alien raccoon (*Procyon lotor*) in the Nopporo Natural Forest Park, Hokkaido, Japan. Assessment and Control of Biological Invasion Risks. 2006; 116–121.

[7] Abe G, Takatsuki S, Yamagiwa J (2008). Raccoon. Mammalogy in Japan.

[8] Freeman PW, Vaughan TA, Ryan JM, Czaplewski NJ (2015). Ecology. Mammalogy.

[9] Kuzmin SL (1995). The problem of food competition in amphibians. Herpetol J..

[10] Blackburn TM, Essl F, Evans T, Hulme PE, Jeschke JM, Kühn I, Kumschick S, Marková Z, Mrugała A, Nentwig W, Pergl J, Pyšek P, Rabitsch W, Ricciardi A, Richardson DM, Sendek A, Vilà M, Wilson JR, Winter M, Genovesi P, Bacher S (2014). A unified classification of alien species based on the magnitude of their environmental impacts. PLOS Biol..

[11] Saeki M, Takatsuki S, Yamagiw J (2008). Raccoon dog. Mammalogy in Japan.

[12] Kim SI, Oshida T, Lee H, Min MS, Kimura J (2015). Evolutionary and biogeographical implications of variation in skull morphology of raccoon dogs (*Nyctereutes procyonoides*, Mammalia: Carnivora). Biol J Linn Soc.

[13] Kim SI, Park SK, Lee H, Oshida T, Kimura J, Kim YJ, Nguyen ST, Sashika M, Min MS (2013). Phylogeography of Korean raccoon dogs: implications of peripheral isolation of a forest mammal in East Asia. J Zool.

[14] Fukue Y, Takeshita T, Nakanishi N (2011). Diet analysis methods to assess the food habits of carnivore in Japan–I. Canidae, Mustelidae. Felidae. Mammal Sci..

[15] Mizukami R, Goto M, Izumiyama S, Yoh M, Ogura N, Hayashi H (2005). Temporal diet changes recorded by stable isotopes in Asiatic black bear (*Ursus thibetanus*) hair. Isot Environ Health Stud.

[16] Deniro MJ (1978). Influence of diet on the distribution of carbon isotopes in animals. Geochim Cosmochim Acta..

[17] Deniro MJ, Epstein S (1981). Influence of diet on the distribution of nitrogen isotopes in animals. Geochim Cosmochim Acta..

[18] Minagawa M, Wada E (1984). Stepwise enrichment of δ^15^N along food chains: further evidence and the relation between δ^15^N and animal age. Geochim Cosmochim Acta.

[19] Tieszen L, Boutton T, Tesdahl K, Slade N (1983). Fractionation and turnover of stable carbon isotopes in animal tissues: implications for δ^13^C analysis of diet. Oecologia.

[20] Hobson K, Clark R (1992). Assessing avian diets using stable isotopes I: turnover of δ^13^C in tissues. Condor..

[21] Hobson K, Clark R (1993). Turnover of δ^13^C in cellular and plasma fractions of blood: implications for nondestructive sampling in avian dietary studies. Auk.

[22] Takatsuki S, Kubozono M, Minami M (2014). Dietary analysis of raccoons captured in Yokohama, eastern Japan. Jpn J Conserv Ecol..

[23] Matsuo R, Ochiai K (2009). Dietary overlap among two introduced and one native sympatric carnivore species, the raccoon, the masked palm civet, and the raccoon dog, Chiba Prefecture, Japan. Mammal Study..

[24] Margalef R (1958). Information theory in ecology. Gener Syst.

[25] Hamilton WJ (1936). The food and breeding habits of the raccoon. Ohio J Sci..

[26] Sonenshine DE, Winslow EL (1972). Contrasts in distribution of raccoons in two Virginia localities. J Wildl Manag.

[27] Turkowski FJ, Mech LD (1968). Radio-tracking the movements of a young male raccoon. J Minn Acad Sci..

[28] Sako T, Kawada S, Tezuka M, Uesugi T, Akihito (2008). Seasonal food habits of the raccoon dog, *Nyctereutes procyonoides*, in the Imperial Palace, Tokyo. Bull Natl Mus Nat Sci Ser A Zool..

[29] Freeman PW, Vaughan TA, Ryan JM, Czaplewski NJ (2015). Behavior. Mammalogy.

[30] Izumiyama S, Nakashita R, Suzuki Y, Kishimoto R, Takii A, Hayashi H (2012). Feeding habit analysis of an Asiatic black bear that intruded into beef cattle barns in Shiojiri by measuring carbon and nitrogen stable isotope ratios. Bull Shinshu Univ Alpine Field Center..

[31] Mizukami RN, Goto M, Izumiyama S, Hayashi H, Yoh M (2005). Estimation of feeding history by measuring carbon and nitrogen stable isotope ratios in hair of Asiatic black bears. Ursus..

[32] Hori S, Matoba Y (2001). Arthropods recognised from the contents in the digestive tract of raccoons. Bull Hist Mus Hokkaido..

[33] Helle E, Kauhala K (1991). Distribution history and present status of the raccoon dog in Finland. Holarct Ecol..

[34] Mcfadden KW, Sambrotto RN, Medellin RA, Gompper ME (2006). Feeding habits of endangered Pygmy Raccoons (*Procyon pygmaeus*) based on stable isotope and fecal analyses. J Mammal.

[35] Ishikawa Y, Ito K (1989). The regeneration process in a mixed forest in central Hokkaido, Japan. Vegetation..

[36] Kadosaki M, Nozawa I (2009). Morphology and ecology of 20 species mammals in Hokkaido. Raccoon. Illustrated wildlife traces.

[37] Kurashima O, Niwase N (1998). Spacing pattern of feral raccoons (*Procyon lotor*) in Eniwa. Hokkaido. Mammal Sci..

[38] Takatsuki S (2013). Applicability of the point-frame method for food habit analysis for various mammals. Mammal Sci..

[39] Pianka ER (1973). The structure of lizard communities. Annu Rev Ecol Syst.

[40] Gotelli NJ, Entsminger GL (2003). Swap algorithms in null model analysis. Ecology.

[41] López-García J, Navia AF, Mejía-Falla PA, Rubio EA (2012). Feeding habits and trophic ecology of *Dasyatis longa* (Elasmobranchii: Myliobatiformes): sexual, temporal and ontogenetic effects. J Fish Biol.

[42] Roth JD, Hobson KA (2000). Stable carbon and nitrogen isotopic fractionation between diet and tissue of captive red fox: implications for dietary reconstruction. Can J Zool..

