# Supplementary material for: Comparison of feeding habits and habitat use between invasive raccoons and native raccoon dogs in Hokkaido, Japan
Source: BMC Ecol. 2019 Sep 11;19:35. doi: 10.1186/s12898-019-0249-5 (PMC6737712; doi:10.1186/s12898-019-0249-5)
Supplement: Supplementary file 1 — Additional file 1. Stable isotope ratios of δ13C and δ15N for potential prey items. [file 12898_2019_249_MOESM1_ESM.docx]

Additional file 1. Stable isotope ratios of δ^13^C and δ^15^N for potential prey items.

Among potential prey items, corn (−10.49‰ ± 0.20‰) and livestock forage crops (−17.90‰ ± 0.12‰) were high in δ^13^C, whereas plants (−31.65‰ ± 0.62‰), wild fruits (−28.15‰ ± 1.12‰), earthworms (−27.33‰ ± 0.22‰), and insects (−26.22‰ ± 1.21‰) were low in δ^13^C. Reptiles (6.31‰ ± 0.07‰), birds (6.08‰ ± 0.04‰), corn (4.70‰ ± 0.33‰), and amphibians (4.33‰ ± 1.84‰) were high in δ^15^N, whereas plants (−1.98‰ ± 1.32‰), wild fruits (−2.13‰ ± 0.33‰), earthworms (0.62‰ ± 0.08‰), and insects (1.50‰ ± 0.98‰) were low in δ^15^N. Gastropods had the lowest δ^15^N in our study (−5.08‰ ± 0.05‰).
