# Supplementary material for: Comparison of feeding habits and habitat use between invasive raccoons and native raccoon dogs in Hokkaido, Japan
Source: BMC Ecol. 2019 Sep 11;19:35. doi: 10.1186/s12898-019-0249-5 (PMC6737712; doi:10.1186/s12898-019-0249-5)
Supplement: Supplementary file 2 — Additional file 2. Information on the process of hair growth. [file 12898_2019_249_MOESM2_ESM.docx]

Additional file 2. Hair growth process.

When stable isotope analysis is conducted, the molting pattern of the animal is important. However, the molting patterns of raccoons and raccoon dogs in Hokkaido were unknown. We observed seasonal changes in hair growth and molting patterns using captured animals, including those captured for nuisance control. Hair roots are black during the growth stage and white when hair growth stops. The hairs of animals were divided into summer coat and winter coat by capturing season and hair length.

From May to mid-July, both species’ winter coats of the previous year remained, and the hair roots were nearly gone. For early-molting individuals, winter coats began to gradually fall out at the end of May, and short summer hair began to grow underneath. All animals, even late-molting individuals, had a complete summer coat by August. After September, the hair roots of summer coats whitened and hair growth stopped. Although winter coats began to grow beneath summer coats in mid-September, it was possible to distinguish the two based on root hair color: summer coat hairs were white and winter coat hairs were black. By December, hair roots of winter coats were white and growth had stopped. The hair length of summer coats was 4–6 cm, whereas the hair length of winter coats was 6–9 cm.

Our observations of molting patterns are consistent with those for raccoons from North America [1]. From the end of May to mid-July, animals had both winter and summer coats (long and short hair, respectively). The winter coat reflected feeding habits during mid-September to November of the previous year, and the summer coat reflected feeding habits from the end of May to the day of capture. We collected hair samples of 93 raccoons: 25 with summer coat (19 from our field study, six from nuisance control) and 68 with winter coat (all from our field study). We also collected hair samples from 86 raccoon dogs: 17 with summer coat (16 from our field study, 1 from a carcass) and 69 with winter coat (all from our field study).
